# Recent Developments in the Theory and Applicability of Swarm Search

**DOI:** 10.3390/e25050710

**Published:** 2023-04-25

**Authors:** Yaniv Altshuler

**Affiliations:** MIT Media Lab, Cambridge, MA 02139-4307, USA; yanival@media.mit.edu

## 1. Overview

Swarm intelligence (SI) is a collective behaviour exhibited by groups of simple agents, such as ants, bees, and birds, which can achieve complex tasks that would be difficult or impossible for a single individual. The collective behaviour of these organisms is characterized by decentralized decision making, self-organization, adaptive responses to environmental changes, and emergent properties that are not present in individual organisms. SI algorithms emulate these features to solve complex optimization, control, classification, clustering, routing, and prediction problems in diverse domains, such as engineering, robotics, biology, economics, social sciences, and humanities [1].

SI algorithms can be classified into two main categories: swarm-based algorithms and swarm-inspired algorithms [2]. Swarm-based algorithms involve the simulation of a population of individuals (agents) that interact with each other and their environment to achieve a collective goal. Examples of swarm-based algorithms include ant colony optimization (ACO) [3], particle swarm optimization (PSO) [4], artificial bee colony (ABC) [5], and firefly algorithm (FA) [6]. Swarm-inspired algorithms, on the other hand, extract specific mechanisms or principles from natural swarms and incorporate them into conventional optimization or machine learning algorithms. Examples of swarm-inspired algorithms include artificial immune systems (AIS) [7], bacterial foraging optimization (BFO) [8], and grey wolf optimizer (GWO) [9].

The success of SI algorithms is attributed to their ability to efficiently explore a large search space, converge to optimal or near-optimal solutions, and handle multiple objectives or constraints simultaneously. The collective intelligence of the swarm enables the sharing and exchange of information, the exploitation of promising regions, and the avoidance of suboptimal regions. Furthermore, the decentralized and distributed nature of the swarm allows for scalability, robustness, fault-tolerance, and adaptivity to dynamic or uncertain environments [10].

Despite their advantages, SI algorithms face several challenges and limitations, such as premature convergence, scalability issues, sensitivity to parameter settings, lack of theoretical guarantees, and difficulty in interpreting or explaining the obtained results. Researchers have proposed various approaches to overcome these challenges, such as hybridization with other optimization or machine learning techniques, dynamic adaptation of parameters, incorporation of domain knowledge, and rigorous analysis of convergence properties.

## 2. Applications

The advancement of technology has spurred a growing demand for multi-agent and swarm robotics solutions to address an ever-expanding range of complex and diverse challenges. With the emergence of distributed systems, it has become increasingly clear that relying solely on a single robot may not be the optimal approach for many application domains. Instead, teams of robots are being called upon to work in a coordinated and intelligent fashion, leveraging the power of redundancy to achieve greater efficiency and reliability.

The benefits of multi-agent systems stem from their ability to harness the collective intelligence of multiple entities, allowing them to tackle complex tasks that would be beyond the capability of a single robot. This approach provides the flexibility to scale up or down the number of robots based on the task at hand, while also providing redundancy to ensure mission success even in the face of individual robot failures. Moreover, multi-agent systems can leverage complementary skills and diverse perspectives, leading to improved problem-solving capabilities and more robust decision making.

Swarm robotics takes the concept of multi-agent systems a step further by drawing inspiration from the collective behaviour of natural swarms, such as ants, bees, and birds. Swarm robotics seeks to emulate the self-organizing and adaptive behaviour of swarms in order to create distributed systems that can operate autonomously and efficiently. By leveraging simple local interactions between agents, swarm robotics can achieve complex global behaviours, such as exploration, foraging, or assembly, without the need for centralized control or explicit communication. The emergence of swarm robotics opens up exciting new possibilities for applications in fields such as search and rescue, environmental monitoring, and precision agriculture.

In [11], a detailed description of swarm-robotics application domains is presented, demonstrating how large-scale decentralized systems of autonomous robotic agents can be significantly more effective than a single robot in many areas. However, when designing such systems it should be noted that simply increasing the number of robots assigned to a task does not necessarily improve the system’s performance—multiple robots must intelligently cooperate to avoid disturbing each other’s activity and achieve efficiency.

In nature, “simple-minded” animals such as ants, bees or birds cooperate to achieve common goals and exhibit amazing feats of collaborative work. It seems that these animals are “programmed” to interact locally in such a way that the desired global behaviour is likely to emerge even if some individuals of the colony die or fail to carry out their task for other reasons. A similar approach may be considered for coordinating a group of robots without a central supervisor, by using only local interactions between the robots. When this decentralized approach is used, much of the communication overhead (typical of centralized systems) is saved, the hardware of the robots can be fairly simple, and better modularity is achieved. A properly designed system should be readily scalable, achieving reliability through redundancy.

There are several key advantages to the use of such intelligent swarm robotics. First, such systems inherently enjoy the benefit of parallelism. In task-decomposable application domains, robot teams can accomplish a given task more quickly than a single robot, by dividing the task into sub-tasks and executing them concurrently. In certain cases, a single robot may simply be unable to accomplish the task on its own (e.g., to carry a large and heavy object).

Second, decentralized systems tend to be, by their very nature, much more robust than centralized systems (or systems comprised of a single but very complex unit). Generally speaking, a team of robots may provide a more robust solution by introducing redundancy, and by eliminating any single point of failure, while considering the alternative of using a single sophisticated robot, we should note that even the most complex and reliable robot may suffer an unexpected malfunction, which will prevent it from completing its task. When using a multi-agent system, on the other hand, even if a large number of the agents stop working for some reason, the entire group will often still be able to complete its task, although perhaps slower. For example, for exploring a hazardous region (such as a minefield or the surface of Mars), the benefit of redundancy and robustness offered by a multi-agent system is quite obvious, and it is in this context that Rodney Brooks wrote their famous “Fast, Cheap and Out of Control” report [12].

Another advantage of the decentralized swarm approach is the ability of dynamically reallocating sub-tasks between the swarm’s units, thus adapting to unexpected changes in the environment. Furthermore, since the system is decentralized, it can respond relatively quickly to such changes, due to the benefit of locality—the ability to swiftly respond to changes without the need of notifying a hierarchical “chain of command”. Note that as the swarm becomes larger, this advantage becomes increasingly important.

In addition to the ability of quick response to changes, the decentralized nature of such systems also improves their scalability. The scalability of multi-agent systems is derived from relying on the “emergence” of task completion by inherently low communication and computation overhead protocol implemented by the agents. As the tasks assigned nowadays to multi-agent-based systems become increasingly complex, so does the importance of the high scalability of the systems.

Finally, by using heterogeneous swarms, even more efficient systems could be designed, thanks to the utilization of different types of agents whose physical properties enable them to perform much more efficiently in certain special tasks.

Significant research effort has been invested during the last few years in the design and simulation of multi-agent robotics and intelligent swarm systems (see, e.g., [13,14,15,16,17,18,19,20]).

Such designs are often inspired by biology (see [21,22] for evolutionary algorithms, [23] or [24,25] for behaviour-based control models, [26,27,28,29] for flocking and dispersing models, [30,31,32] for predator–prey approaches and [33] for models inspired by the behaviour of cats), by physics [34,35,36], sociology [37,38,39], network theory [40,41,42,43] or by economics applications [44,45,46,47,48,49,50,51,52,53,54].

A swarm-based robotics system can generally be defined as a highly decentralized group of extremely simple robotic agents, with limited communication, computation and sensing abilities, designed and deployed to accomplish various tasks. Tasks that have been of particular interest to researchers in recent years include synergetic mission planning [55,56], emergency detection using decentralized sensing capabilities [57], patrolling [58,59,60], fault tolerance cooperation [61,62,63], network security [64], adversarial learning modelling [65], financial system modelling [66], crowd modelling [67], swarm control [68,69], human design of mission plans [70,71], role assignment [72,73,74,75,76], multi-robot path planning [59,77,78,79,80,81], traffic control [82,83,84], formation generation [85,86,87,88], formation keeping [89,90,91], exploration and mapping [45,92,93], target tracking [94,95], collaborative cleaning [96,97,98,99], control architecture for autonomous drones [100,101] and target search [102,103].

Unfortunately, the mathematical and geometrical theory of such multi-agent systems is far from being satisfactory, as pointed out in [104,105,106,107] and many other papers.

Our interest is focused on developing the mathematical tools necessary to design and analyse such systems. For example, in [108] it was shown that a number of agents can arrange themselves equidistantly in a row via a sequence of linear adjustments, based on a simple “local” interaction. The convergence of the configuration to the desired one is exponentially fast. A different way of cooperation between agents, inspired by the behaviour of ant colonies, is described in [109]. There it was proven that a sequence of ants engaged in deterministic chain pursuit will find the shortest (i.e., straight) path from the ant hill to the food source, using only local interactions. In [110], the behaviour of a group of agents on $Z^{2}$ was investigated, where each ant-like agent pursued their predecessor, according to a discrete biased-random-walk model of pursuit on the integer grid. The average paths of such a sequence of a(ge)nts engaged in a chain of probabilistic pursuit was shown to converge to the “straight line” between the origin and destination, and this too happens exponentially fast.

An in-depth analysis of the effect of certain geometric properties on the search efficiency of a collaborative swarm of autonomous drones appears in [111,112], whereas an example of a set of analytic complexity bounds for this problem can be found in [113,114]. A work that analysed the effect of a stochastic framework for the same problem is presented in [115].

## 3. Decentralized Intelligence Architectures and the Swarm Paradigm

A key principle in the notion of swarms, or multi-agent robotics, is the simplicity of the individual agent. The notion of “simplicity” here means that the agents should be significantly simpler than a “single sophisticated system”, which can be constructed for the same purpose. As a result, the capabilities and the resources of such simple agents are assumed to be very limited, with respect to the following aspects:

- Memory resources—basic agents should be assumed to contain only O(1) memory resources (i.e., the size of memory is independent of the size of the problem or the number of agents). This usually imposes many interesting limitations on the agents. For example, agents can remember only a limited history of their activities so far. Thus, protocols designed for agents with such limited memory resources are usually very simple and attempt to solve a given problem by relying on some (necessarily local) basic patterns arising in the environment. The task is completed by a repetition of these patterns by a large number of agents.
- Sensing capabilities—defined according to the specific nature of the problem. For example, for agents moving along a 100×100 grid, a reasonable sensing radius may be 3 or 4, but certainly not 40.
- Computational resources—although agents are assumed to employ only limited computational resources, a formal definition of this constraint is hard to define. In general, most of the time-polynomial algorithms may be used, provided that the amount of memory the agents have is sufficient.
- Communication is very limited—the issue of communication in multi-agent systems has been extensively studied in recent years. Distinctions between implicit and explicit communication are usually made, in which implicit communication occurs as a side effect of other actions, or “through the world” (see, for example [116]), whereas explicit communication is a specific act intended solely to convey information to other robots on the team. Explicit communication can be performed in several ways, such as a short-range point-to-point communication, a global broadcast, or by using some sort of distributed shared memory. Such memory is often referred to as a pheromone, used to convey small amounts of information between the agents [22,117,118,119]. This approach is inspired from the coordination and communication methods used by many social insects—studies on ants (e.g., [120,121]) show that the pheromone-based search strategies used by ants in foraging for food in unknown terrains tend to be very efficient. Additional information can be found in the relevant NASA survey, focusing on “intelligent swarms” comprised of multiple “stupid satellites” [122,123] or the following survey conducted by the US Naval Research Center [124]. The lack of explicit communication poses an challenge for various special configuration sets, such as symmetric environments [111].
  In the spirit of designing a system which uses as simple agents as possible, we aspire that the agents will have as little communication capabilities as possible. With respect to the taxonomy of multi-agents discussed in [125], we would be interested in using agents of the types COM-NONE or if necessary COM-NEAR with respect to their communication distances, and BAND-MOTION, BAND-LOW or even BAND-NONE (if possible) with respect to their communication bandwidth. Therefore, although a certain amount of implicit communication can hardly be avoided (due to the simple fact that by changing the environment, the agents are constantly generating some kind of implicit information), explicit communication should be strongly limited or avoided altogether, in order to fit our paradigm (note that in many works in this field, this is not the case, and communication, as well as memory, resources, are often being used in order to create complex cooperative systems).

In summary, while designing intelligent swarm systems we must assume (and often even aspire for) having an available individual agents that are myopic, mute, senile and rather stupid.

## 4. Limitations

While SI has been applied successfully in many fields, including optimization, robotics, and networking, it also has limitations that need to be taken into account. One of the main limitations of SI is its sensitivity to initial conditions and parameter settings. Small changes in the initial configuration or the parameters of the swarm can have a significant impact on its behaviour and performance, leading to suboptimal solutions or even failure to converge. This problem is exacerbated in large-scale systems, where the number of variables and interactions increases exponentially [10].

Another limitation of SI is its vulnerability to perturbations and disturbances. Swarms are designed to be robust and resilient to individual failures or disruptions, but they can be vulnerable to systemic disturbances [126], such as environmental changes, resource depletion, or external attacks. These disturbances can destabilize the swarm beyond its self-emergent macroscopic regularities [127], leading to disintegration, divergence, or oscillations.

Real-world examples of these limitations include the behaviour of ant colonies in changing environments. Ants use SI to forage for food and build nests, but they are also susceptible to disturbances such as climate change or human intervention. In some cases, ant colonies can collapse or become maladapted to their environment due to the loss of critical resources or the disruption of communication channels.

Another limitation of SI is related to the trade-off between exploration and exploitation. Swarms can achieve impressive results by exploring a large search space and exploiting the best solutions found. However, there is a risk of getting stuck in local optima or suboptimal regions of the search space, especially if the swarm lacks diversity or adaptability [128]. In some cases, the swarm may require a balance between exploration and exploitation to achieve the best results, which can be challenging to achieve in practice [54].

A related limitation is the scalability of SI [113], while swarms can scale up to thousands or millions of agents, the computational and communication overheads can become prohibitive in large-scale systems. The swarm may require efficient algorithms for coordination, decision making, and resource allocation, which can be difficult to design and optimize. Such limitations may take form, for example, when SI is used in traffic management systems. Swarms of autonomous vehicles or drones can optimize traffic flow and reduce congestion by coordinating their movements and avoiding collisions [83]. However, these systems require efficient algorithms for path planning, decision making, and communication, as well as robust mechanisms for handling uncertainties and unexpected events.

Another example is the application of SI in social networks. Swarms of agents can learn and adapt to social dynamics by interacting with each other and with the environment [129]. However, these systems are also susceptible to biases, echo chambers, and polarization, which can affect their ability to explore new ideas and perspectives [130].

## 5. Swarm Search with Communication

While decentralized swarms have been the main focus of swarm-based search algorithms due to their scalability and simplicity, there are also several promising works that utilize synchronization or communication among the agents. These parallel swarms often employ communication to enhance the efficiency of the search process, such as parallel ant colony optimization, parallel particle swarm optimization, and other parallel metaheuristic approaches.

Parallel ant colony optimization (PACO) [131] is an example of a parallel swarm algorithm that utilizes communication among agents. PACO algorithms allow multiple agents to cooperate by sharing pheromone information, which helps in quickly identifying the optimal solution. For instance, PACO has been used in multi-robot coverage problems, where a group of robots are required to explore an unknown environment while avoiding collisions with each other. By sharing pheromone information, the robots can quickly converge to a solution, even in complex and large environments [22,132].

Parallel particle swarm optimization (PPSO) [133] is another example of a parallel swarm algorithm that uses communication among agents. PPSO is a variant of particle swarm optimization (PSO) that allows multiple agents to communicate with each other to improve the search process. For instance, PPSO has been used to optimize complex systems such as power grids, where the agents need to communicate to efficiently manage the distributed resources [134].

In cases where decentralized swarms may not be sufficient, parallel swarms can be beneficial. For example, in situations where the problem space is complex, the search space is vast, and the search process is time-critical, parallel swarm algorithms can offer a significant advantage over decentralized swarms. In such scenarios, communication among agents can help to identify the optimal solution more quickly and efficiently.

However, one of the main drawbacks of parallel swarm algorithms is the increased complexity of the communication mechanisms, which may require significant computational resources [135]. Additionally, communication can also lead to increased synchronization overhead, which may impact the scalability of the algorithm. Thus, in cases where the problem space is relatively simple, decentralized swarm algorithms may still be a better choice.

While decentralized swarms remain the main focus of swarm-based search algorithms, parallel swarm algorithms that utilize communication among agents have shown significant promise in enhancing the efficiency of the search process. These algorithms have been used in various applications, such as multi-robot coverage problems and power grid optimization. However, the increased complexity of communication mechanisms and synchronization overhead should also be considered when deciding on the appropriate approach for a given problem.

## 6. Opportunities and Future Research

SI and swarm systems have received considerable attention in recent years due to their potential for solving complex problems in various fields, such as robotics, optimization, and network design. As a result, there are numerous opportunities for future research in this area.

One promising avenue for future research is the development of more sophisticated algorithms and models for SI, while current approaches have shown promise, there is still much to be done in terms of improving the efficiency and adaptability of swarm systems [136]. Researchers may explore new ways to optimize the communication and coordination of swarm agents, or develop new approaches for dealing with the inherent uncertainty and complexity of real-world environments [137].

Another important area for future research is the application of SI to real-world problems, while there have been many successful demonstrations of swarm systems in laboratory settings, there is a need for more research on how to apply these systems to real-world problems. This may involve working with industry partners to develop practical solutions that can be deployed in the field, or collaborating with government agencies to address societal challenges such as disaster response or urban planning [138,139].

In addition to these technical challenges, there are also important ethical and social considerations to be addressed. As swarm systems become more advanced and pervasive, there may be concerns around issues such as privacy, security, and control. Researchers may need to explore new ways to address these concerns, such as developing transparent and accountable algorithms, or working with policymakers to establish appropriate regulations and standards [140].

Overall, there are numerous opportunities for future research in SI and swarm systems. By continuing to explore these systems and their potential applications, researchers can help to unlock new solutions to complex problems and contribute to the advancement of science and technology.

## 7. Conclusions

The study of SI has revealed that even seemingly simple organisms, such as ants, can exhibit complex and sophisticated collective behaviours when allowed to work together in a synergistic manner. This insight has led researchers to investigate the potential for applying this approach to artificial intelligence and robotics, with promising results.

In this Special Issue, a number of research studies have been presented that demonstrate the power of SI in producing complex and adaptive behaviours. By studying the ways in which ants and other social insects cooperate and communicate with one another, researchers have been able to develop algorithms and models that can be applied to a wide range of problems.

One of the key insights from these studies is that individual agents within a swarm do not necessarily need to be highly intelligent or even aware of the larger goals of the group. Rather, by following simple rules and responding to local cues, they can collectively produce intelligent and adaptive behaviours that emerge at the swarm level.

This approach has numerous potential applications, from optimizing traffic flow to coordinating the movements of swarms of robots in search and rescue operations. By harnessing the power of SI, researchers are exploring new ways to tackle complex problems that would be difficult or impossible for any individual agent to solve alone.

Overall, the research presented in this Special Issue provides compelling evidence that even the simplest organisms can exhibit remarkable intelligence and adaptability when working together in a synergistic manner. By taking inspiration from nature, researchers are opening up exciting new avenues for developing advanced technologies that can benefit society in countless ways.

In summary, let us cite a statement made by a scientist after watching an ant making his laborious way across a wind-and-wave-moulded beach [141]:

“An ant, viewed as a behaving system, is quite simple. The apparent complexity of its behavior over time is largely a reflection of the environment in which it finds itself.”

Such a point of view, as well as the results of the research presented in this Special Issue, lead us to believe that even simple, ant-like beings, when allowed to synergically collaborate, can yield a complicated, adaptive and quite efficient macroscopic behaviour, in the intelligent swarm-level scope.
